# Literary Notes

**Published:** 1909-07-17

**Authors:** 


### LITERARY NOTES.

### REPORT OF THE LOCAL GOVERNMENT BOARD INSPECTORS OF FOODS.

We have received a report by Dr. G. S. Buchanan, of the Local Government Board, summarising the work done in the new department of the Board devoted to the inspection of food, which has been in existence for two years. The department now consists of an inspector and two assistant inspectors, outside technical assistance being co-opted when required. The food substances dealt with are tartaric acid, vinegar, imported meat foods, and canned and tinned meats. In addition to this, the questions of preservatives, food inspection at English ports, the Public Health (Regulations as to Food) Act, 1907, and foreign and colonial food legislation are discussed. The report is distinctly interesting, and shows that the department in question is, while being active in the interest of the public health, not hypercritical or cantankerous with regard to the great food industries. A separate leaflet contains the report of Dr. J. M. Hamill concerning the "facing" and other methods of preparing rice for sale. From this extremely interesting report it appears that both during the process of milling and subsequently for the purpose of polishing or glazing, rice is treated with talc to such an extent that it is not uncommon for the finished article to contain as much as 2 per cent. of extraneous mineral matter. In addition, for the purpose of producing a dead white appearance in the grain, certain blue dyes are also called into requisition. There is no suggestion in Dr. Hamill's report that these substances in the quantity likely to be consumed are injurious to health, but he does suggest that a rice containing more than 0.5 per cent. of mineral matter is essentially to the prejudice of the consumer in the sense of the Sale of Food and Drugs Act, in that the mineral matter in question is cheaper than the original rice and detracts from its nutritive value.

In current exegetical literature, we believe, the bibliography appended to an article is reckoned almost as important as the article itself. Things have not got this length yet in medicine. Rare studies there are, so original in conception as to have no relation to previous work, while a paper with anything in it cannot be spoilt by the omission of references, although these may well form the *differentia* between merely a good paper and an excellent one. A publication solely bibliographical in scope which fulfils its purpose very well is the *Internationales Centralblatt für die gesamte Tuberkulose-Forschung.* From the current number of this we learn of a statement made at the thirtieth meeting of Balneologists in Berlin, which will cause surprise in a considerable portion of the medical public of this country. It is that Sir Almroth Wright has long convinced himself of the impracticability (*Undurchführbarkeit*) of opsonin estimation in tuberculous subjects, and that in his clinic dosage and timing of tuberculin injections are settled from general clinical observation. Although the title of the article runs "On the necessity of opsonic control . . ."—the writer does not show himself in this matter more royalist than the King.

